# Bis[*N*,*N*-bis­(diphenyl­phosphan­yl)cyclo­hexyl­amine-κ^2^ *P*,*P*′]platinum(II) bis­(hexa­fluorido­phosphate) dichloro­methane disolvate

**DOI:** 10.1107/S1600536810028795

**Published:** 2010-07-24

**Authors:** Ilana Engelbrecht, Hendrik G. Visser, Andreas Roodt

**Affiliations:** a Department of Chemistry, University of the Free State, PO Box 339, Bloemfontein 9300, South Africa

## Abstract

In the title compound, [Pt(C_30_H_31_NP_2_)_2_](PF_6_)_2_·2CH_2_Cl_2_, the four-coordinated Pt^II^ atom, situated on an inversion centre, exhibits a highly distorted square-planar geometry illustrated by the P—Pt—P bite angle of 70.76 (3)°. The cyclo­hexyl ring and one of the phenyl rings display 0.630 (7):0.37 (7) and 0.60 (2):0.40 (2) positional disorder, respectively. The dichloro­methane solvent mol­ecule displays 0.526 (4):0.474 (4) positional disorder. C—H⋯F hydrogen bonds stabilize the crystal packing.

## Related literature

For applications of Pt(II) diphosphinoamine complexes in homogeneous catalysis, see: Brink *et al.* (2010 ▶); Otto *et al.* (1998 ▶); Roodt & Steyn (2000 ▶); Steyn *et al.* (1992 ▶, 1997 ▶, 2008 ▶); Viljoen *et al.* (2008 ▶, 2009*a* ▶,*b* ▶, 2010 ▶). For related platinum(II) complexes, see: Cloete *et al.* (2010 ▶); Dyson *et al.* (2004 ▶); Engelbrecht *et al.* (2010 ▶); Farrar & Browning (1995 ▶). For related diphenyl­phosphino ligands, see: Cloete *et al.* (2008 ▶, 2009 ▶); Cotton *et al.* (1996 ▶); Fei *et al.* (2003 ▶); Keat *et al.* (1981 ▶).
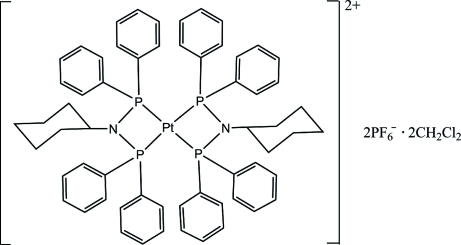


## Experimental

### 

#### Crystal data

[Pt(C_30_H_31_NP_2_)_2_](PF_6_)_2_·2CH_2_Cl_2_
*M* *_r_* = 1588.38
Monoclinic, 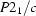

*a* = 13.350 (5) Å
*b* = 18.764 (4) Å
*c* = 15.248 (5) Å
β = 123.333 (5)°
*V* = 3191.3 (17) Å^3^
*Z* = 2
Mo *K*α radiation
μ = 2.59 mm^−1^
*T* = 100 K
0.32 × 0.29 × 0.14 mm

#### Data collection

Bruker X8 APEXII KappaCCD diffractometer
Absorption correction: multi-scan (*SADABS*; Bruker, 2001 ▶) *T*_min_ = 0.491, *T*_max_ = 0.713
57085 measured reflections
7899 independent reflections
6367 reflections with *I* > 2σ(*I*)
*R*_int_ = 0.042

#### Refinement

*R*[*F* ^2^ > 2σ(*F* ^2^)] = 0.027
*wR*(*F* ^2^) = 0.070
*S* = 1.03
7899 reflections
503 parameters
18 restraints
H-atom parameters constrained
Δρ_max_ = 0.72 e Å^−3^
Δρ_min_ = −0.99 e Å^−3^

### 

Data collection: *APEX2* (Bruker, 2007 ▶); cell refinement: *SAINT-Plus* (Bruker, 2007 ▶); data reduction: *SAINT-Plus*; program(s) used to solve structure: *SHELXS97* (Sheldrick, 2008 ▶); program(s) used to refine structure: *SHELXL97* (Sheldrick, 2008 ▶); molecular graphics: *DIAMOND* (Brandenburg & Putz, 1999 ▶); software used to prepare material for publication: *WinGX* (Farrugia, 1999 ▶).

## Supplementary Material

Crystal structure: contains datablocks global, I. DOI: 10.1107/S1600536810028795/hy2330sup1.cif

Structure factors: contains datablocks I. DOI: 10.1107/S1600536810028795/hy2330Isup2.hkl

Additional supplementary materials:  crystallographic information; 3D view; checkCIF report

## Figures and Tables

**Table 1:** Selected bond lengths (Å)

Pt1—P1	2.2918 (9)
Pt1—P2	2.2999 (9)

**Table 2:** Hydrogen-bond geometry (Å, °)

*D*—H⋯*A*	*D*—H	H⋯*A*	*D*⋯*A*	*D*—H⋯*A*
C12*A*—H12*A*⋯F2	0.95	2.45	3.186 (11)	134
C32—H32⋯F2	0.95	2.47	3.138 (4)	127

**Fig. 1.**
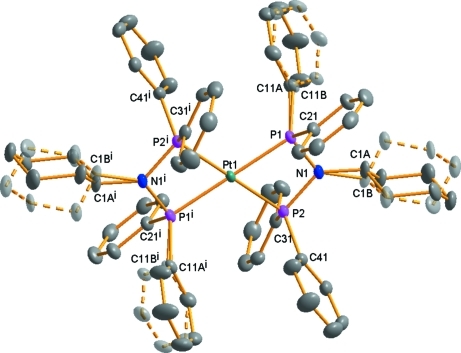
Molecular structure of the complex cation in the title compound. Displacement ellipsoids are drawn at the 50% probability level. Dashed lines denote the bonds between the minor disordered atoms. H atoms, solvent molecules and hexafluorophosphate anions have been omitted for clarity. [Symmetry code: (i) 1-x, 1-y, 1-z.]

**Fig. 2.**
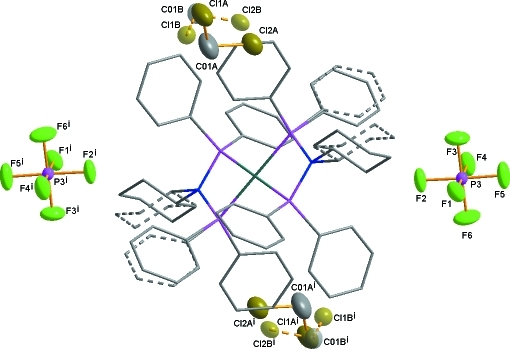
A view of the solvent molecules and hexafluorophosphate anions. Displacement ellipsoids are drawn at the 50% probability level. H atoms have been omitted for clarity. [Symmetry code: (i) 1-x, 1-y, 1-z.]

**Fig. 3.**
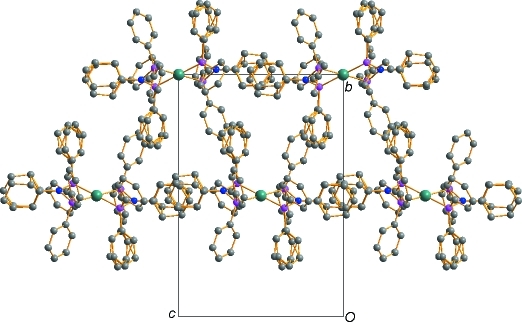
Perspective view of the unit cell of the title compound along the a axis. H atoms, solvent molecules and hexafluorophosphate anions have been omitted for clarity.

## supplementary crystallographic information

### Comment

The synthesis of Pt(II) diphosphinoamine complexes forms part of ongoing research in our group in the field of homogeneous catalysis (Brink *et al.*, 2010; Otto *et al.*, 1998; Roodt & Steyn, 2000; Steyn *et al.*, 1992, 1997, 2008; Viljoen *et al.*, 2008, 2009*a*,*b*, 2010). In the title compound (Fig. 1, Table 1), the Pt^II^ atom is situated on an inversion centre. The strain in the complex is illustrated by the distorted square-planar geometry around the Pt atom, with a P—Pt—P bite angle of 70.76 (3)°, forcing the P1–N1–P2 angle to 102.58 (12)° and demonstrating the deviation from the ideal tetrahedral geometry of the N atom. The N atom is displaced by 0.166 (3) and -0.081 (3) Å from the C1A, P1, P2 and C1B, P1, P2 planes respectively, showing that the N atom adopts an almost planar geometry with the two P atoms and the C atom attached to it to accomodate the steric bulk of the phenyl groups and the cyclohexyl ring of the ligand. The P atoms are also severely distorted from the expected tetrahedral configuration with Pt1—P1—N1 and Pt1—P2—N1 angles being 93.47 (8) and 93.08 (9)°, respectively. The cyclohexyl ring, bonded to the N atom, and the C11-phenyl ring, bonded to P1, display 0.63:0.37 and 0.60:0.40 positional disorder, respectively. The dichloromethane molecules display 0.53:0.47 positional disorder (Fig. 2). Molecules of the title compound pack in horizontal rows across the *bc* plane in the unit cell (Fig. 3). Intermolecular hydrogen bonds exist between C12A—H12A and F2 and C32—H32 and F2 (Table 2).

### Experimental

Pt(cod)Cl_2_ (20 mg, 0.054 mmol) (cod = 1,5-cyclooctadiene) dissolved in a minimum amount of dichloromethane was added in a rapid dropwise manner to a solution of bis(diphenylphosphino)cyclohexylamine (52.5 mg, 0.112 mmol) and NaPF_6_ (19.8 mg, 0.118 mmol) dissolved in a minimum volume of dichloromethane/methanol (1:1). After stirring for 20 min, the solvent was removed completely under reduced pressure. Dichloromethane was added until no further dissolution of solid was evident. The resulting heterogeneous mixture was filtered to remove the insoluble NaCl by-product. The colourless solid product was precipitated upon addition of methanol followed by a reduction in solvent volume under reduced pressure. The compound was isolated by filtration and washed with diethyl ether (10 cm^3^). Layering of a dichloromethane solution of the product with methanol gave colourless crystals, suitable for X-ray diffraction (crude yield: 44 mg, 73%). Spectroscopy data: ^1^H NMR (600 MHz, CD_2_Cl_2_): δ = 0.6 to 1.4 (m, 18H), 3.1 (m, 2H), 3.6 (m, 2H), 7.4 to 7.7 (m, 20H). ^31^P NMR (243 MHz, CD_2_Cl_2_): δ = 39.9 (t, ^1^J_Pt—P_ = 1062.0 Hz), -135.2 to -152.7 (m, PF_6_).

### Refinement

H atoms were positioned geometrically and refined as riding atoms, with C—H = 0.95 (aromatic), 1.00 (CH) and 0.99 (CH_2_) Å and *U*_iso_(H) = 1.2*U*_eq_(C). The highest residual peak is located 0.15 Å from CL1A and the deepest hole is situated 0.56 Å from CL1B.

### Crystal data

**Table:**

[Pt(C_30_H_31_NP_2_)_2_](PF_6_)_2_·2CH_2_Cl_2_	*F*(000) = 1589
*M**_r_* = 1588.38	*D*_x_ = 1.653 Mg m^−^^3^
Monoclinic, *P*2_1_/*c*	Mo *K*α radiation, λ = 0.71069 Å
Hall symbol: -P 2ybc	Cell parameters from 9230 reflections
*a* = 13.350 (5) Å	θ = 2.8–28.2°
*b* = 18.764 (4) Å	µ = 2.59 mm^−^^1^
*c* = 15.248 (5) Å	*T* = 100 K
β = 123.333 (5)°	Cuboid, colourless
*V* = 3191.3 (17) Å^3^	0.32 × 0.29 × 0.14 mm
*Z* = 2	

### Data collection

**Table:**

Bruker X8 APEXII KappaCCD diffractometer	7899 independent reflections
Radiation source: fine-focus sealed tube	6367 reflections with *I* > 2σ(*I*)
graphite	*R*_int_ = 0.042
ω and φ scans	θ_max_ = 28.4°, θ_min_ = 4.2°
Absorption correction: multi-scan (*SADABS*; Bruker, 2001)	*h* = −13→17
*T*_min_ = 0.491, *T*_max_ = 0.713	*k* = −25→24
57085 measured reflections	*l* = −20→20

### Refinement

**Table:**

Refinement on *F*^2^	Primary atom site location: structure-invariant direct methods
Least-squares matrix: full	Secondary atom site location: difference Fourier map
*R*[*F*^2^ > 2σ(*F*^2^)] = 0.027	Hydrogen site location: inferred from neighbouring sites
*wR*(*F*^2^) = 0.070	H-atom parameters constrained
*S* = 1.03	*w* = 1/[σ^2^(*F*_o_^2^) + (0.0297*P*)^2^ + 4.9393*P*] where *P* = (*F*_o_^2^ + 2*F*_c_^2^)/3
7899 reflections	(Δ/σ)_max_ = 0.003
503 parameters	Δρ_max_ = 0.72 e Å^−^^3^
18 restraints	Δρ_min_ = −0.99 e Å^−^^3^

### Special details

**Table:**

Experimental. The intensity data was collected on a Bruker X8 ApexII 4 K Kappa CCD diffractometer using an exposure time of 5 s/frame. A total of 1847 frames were collected with a frame width of 0.5° covering up to θ = 28.36° with 99.1% completeness accomplished.

### Fractional atomic coordinates and isotropic or equivalent isotropic displacement parameters (Å^2^)

**Table:**

	*x*	*y*	*z*	*U*_iso_*/*U*_eq_	Occ. (<1)
C1A	0.7123 (8)	0.5220 (6)	0.8424 (9)	0.0219 (18)	0.630 (7)
H1A	0.6699	0.5599	0.8562	0.026*	0.630 (7)
C2A	0.8441 (5)	0.5445 (3)	0.8991 (3)	0.0299 (14)	0.630 (7)
H2A1	0.8889	0.509	0.8856	0.036*	0.630 (7)
H2A2	0.8497	0.5911	0.8715	0.036*	0.630 (7)
C3A	0.8998 (7)	0.5504 (4)	1.0172 (5)	0.0391 (16)	0.630 (7)
H3A1	0.861	0.59	1.0308	0.047*	0.630 (7)
H3A2	0.9861	0.5618	1.0529	0.047*	0.630 (7)
C4A	0.8855 (6)	0.4809 (3)	1.0631 (4)	0.0405 (17)	0.630 (7)
H4A1	0.9324	0.4426	1.0572	0.049*	0.630 (7)
H4A2	0.9178	0.4879	1.1385	0.049*	0.630 (7)
C5A	0.7558 (9)	0.4589 (5)	1.0064 (8)	0.036 (2)	0.630 (7)
H5A1	0.7105	0.494	1.02	0.043*	0.630 (7)
H5A2	0.7496	0.4119	1.0327	0.043*	0.630 (7)
C6A	0.7028 (19)	0.4544 (9)	0.8898 (17)	0.0228 (17)	0.630 (7)
H6A1	0.7439	0.4159	0.877	0.027*	0.630 (7)
H6A2	0.6172	0.4412	0.8536	0.027*	0.630 (7)
C1B	0.7520 (13)	0.5190 (12)	0.8490 (17)	0.024 (3)	0.370 (7)
H1B	0.831	0.5048	0.8615	0.029*	0.370 (7)
C2B	0.7677 (8)	0.5875 (5)	0.9006 (6)	0.028 (2)	0.370 (7)
H2B1	0.6897	0.6044	0.8862	0.034*	0.370 (7)
H2B2	0.798	0.6234	0.873	0.034*	0.370 (7)
C3B	0.8569 (9)	0.5787 (6)	1.0188 (7)	0.029 (2)	0.370 (7)
H3B1	0.9367	0.5673	1.0325	0.035*	0.370 (7)
H3B2	0.8637	0.6245	1.0539	0.035*	0.370 (7)
C4B	0.8217 (10)	0.5209 (6)	1.0662 (7)	0.040 (3)	0.370 (7)
C5B	0.7999 (16)	0.4511 (10)	1.0077 (15)	0.038 (4)	0.370 (7)
H5B1	0.773	0.4151	1.0379	0.045*	0.370 (7)
H5B2	0.8771	0.4344	1.0202	0.045*	0.370 (7)
C6B	0.705 (4)	0.455 (2)	0.886 (3)	0.040 (3)	0.370 (7)
H6B1	0.624	0.4643	0.8699	0.048*	0.370 (7)
H6B2	0.7037	0.4094	0.8515	0.048*	0.370 (7)
C11A	0.509 (3)	0.6467 (9)	0.648 (2)	0.0207 (12)	0.60 (2)
C16A	0.3952 (15)	0.6778 (12)	0.594 (2)	0.0273 (8)	0.60 (2)
H16A	0.3261	0.6484	0.5654	0.033*	0.60 (2)
C15A	0.3821 (12)	0.7511 (12)	0.5829 (14)	0.030 (3)	0.60 (2)
H15A	0.3045	0.7719	0.5443	0.035*	0.60 (2)
C14A	0.4835 (9)	0.7935 (5)	0.6283 (11)	0.040 (2)	0.60 (2)
H14A	0.4757	0.8439	0.6231	0.048*	0.60 (2)
C13A	0.5958 (7)	0.7629 (4)	0.6811 (13)	0.048 (4)	0.60 (2)
H13A	0.6649	0.7925	0.7121	0.058*	0.60 (2)
C12A	0.6090 (9)	0.6895 (5)	0.6894 (11)	0.036 (2)	0.60 (2)
H12A	0.6866	0.6687	0.7233	0.044*	0.60 (2)
C11B	0.506 (5)	0.6450 (13)	0.655 (3)	0.0207 (12)	0.40 (2)
C12B	0.6086 (14)	0.6845 (7)	0.7260 (12)	0.022 (2)	0.40 (2)
H12B	0.6837	0.6611	0.7682	0.027*	0.40 (2)
C13B	0.6009 (9)	0.7572 (5)	0.7357 (13)	0.030 (3)	0.40 (2)
H13B	0.67	0.7839	0.7849	0.036*	0.40 (2)
C14B	0.4914 (12)	0.7906 (6)	0.6727 (14)	0.028 (3)	0.40 (2)
H14B	0.4861	0.8408	0.6766	0.034*	0.40 (2)
C15B	0.390 (2)	0.7519 (18)	0.604 (2)	0.030 (3)	0.40 (2)
H15B	0.3142	0.7751	0.5647	0.035*	0.40 (2)
C16B	0.398 (2)	0.6792 (19)	0.594 (3)	0.0273 (8)	0.40 (2)
H16B	0.3287	0.653	0.5445	0.033*	0.40 (2)
C21	0.4170 (3)	0.51739 (15)	0.6806 (2)	0.0222 (6)	
C22	0.4104 (3)	0.55086 (17)	0.7594 (2)	0.0279 (7)	
H22	0.4558	0.5927	0.7923	0.034*	
C23	0.3373 (3)	0.52266 (19)	0.7891 (3)	0.0335 (7)	
H23	0.3336	0.5449	0.8432	0.04*	
C24	0.2697 (3)	0.46216 (18)	0.7400 (2)	0.0312 (7)	
H24	0.2199	0.443	0.7608	0.037*	
C25	0.2743 (3)	0.42946 (17)	0.6607 (2)	0.0276 (7)	
H25	0.2265	0.3886	0.6263	0.033*	
C26	0.3486 (3)	0.45657 (16)	0.6320 (2)	0.0241 (6)	
H26	0.3532	0.4335	0.5788	0.029*	
C31	0.8129 (3)	0.50637 (15)	0.6510 (2)	0.0208 (6)	
C32	0.8465 (3)	0.57719 (16)	0.6751 (2)	0.0286 (7)	
H32	0.8087	0.6067	0.6993	0.034*	
C33	0.9353 (3)	0.60518 (18)	0.6641 (3)	0.0377 (8)	
H33	0.9593	0.6535	0.6821	0.045*	
C34	0.9887 (3)	0.56276 (18)	0.6270 (3)	0.0333 (7)	
H34	1.0486	0.5821	0.6184	0.04*	
C35	0.9553 (3)	0.49219 (17)	0.6021 (3)	0.0300 (7)	
H35	0.9919	0.4633	0.5762	0.036*	
C36	0.8685 (3)	0.46348 (16)	0.6149 (2)	0.0234 (6)	
H36	0.8469	0.4147	0.5992	0.028*	
C41	0.7127 (3)	0.38095 (14)	0.6872 (2)	0.0215 (6)	
C42	0.8280 (3)	0.35531 (16)	0.7592 (2)	0.0255 (6)	
H42	0.8952	0.3859	0.7847	0.031*	
C43	0.8445 (3)	0.28564 (17)	0.7933 (2)	0.0317 (7)	
H43	0.923	0.2683	0.8424	0.038*	
C44	0.7477 (4)	0.24134 (18)	0.7563 (3)	0.0406 (9)	
H44	0.7593	0.1935	0.7805	0.049*	
C45	0.6336 (4)	0.26580 (19)	0.6842 (3)	0.0455 (9)	
H45	0.567	0.2346	0.6585	0.055*	
C46	0.6156 (3)	0.33545 (16)	0.6491 (3)	0.0321 (7)	
H46	0.5369	0.3521	0.599	0.039*	
N1	0.6583 (2)	0.52112 (12)	0.72778 (17)	0.0206 (5)	
F1	1.0498 (2)	0.69802 (10)	0.96793 (16)	0.0490 (6)	
F2	0.8895 (2)	0.71702 (11)	0.80417 (17)	0.0544 (6)	
F3	0.9056 (2)	0.77212 (16)	0.9418 (2)	0.0720 (8)	
F4	0.9237 (2)	0.83448 (11)	0.82524 (17)	0.0555 (6)	
F5	1.0843 (2)	0.81596 (12)	0.98976 (18)	0.0632 (7)	
F6	1.0704 (2)	0.76089 (16)	0.8535 (2)	0.0741 (8)	
P1	0.51549 (7)	0.55043 (4)	0.64387 (5)	0.01807 (14)	
P2	0.68416 (6)	0.47263 (4)	0.64749 (5)	0.01702 (14)	
P3	0.98835 (7)	0.76603 (4)	0.89695 (6)	0.02531 (17)	
Pt1	0.5	0.5	0.5	0.01390 (5)	
Cl1A	0.2941 (3)	0.24968 (16)	1.0253 (3)	0.0898 (11)	0.526 (4)
Cl2A	0.4137 (3)	0.36092 (12)	0.98377 (18)	0.0645 (8)	0.526 (4)
C01A	0.3384 (12)	0.2783 (6)	0.9426 (8)	0.088 (4)	0.526 (4)
H01A	0.2669	0.283	0.8701	0.105*	0.526 (4)
H01B	0.3921	0.2422	0.9419	0.105*	0.526 (4)
Cl1B	0.3117 (2)	0.20374 (13)	1.00257 (18)	0.0514 (8)*	0.474 (4)
Cl2B	0.3439 (3)	0.35456 (12)	0.9852 (2)	0.0534 (7)	0.474 (4)
C01B	0.2399 (8)	0.2844 (4)	0.9415 (7)	0.049 (2)	0.474 (4)
H01C	0.1982	0.2794	0.8644	0.058*	0.474 (4)
H01D	0.179	0.2956	0.9574	0.058*	0.474 (4)

### Atomic displacement parameters (Å^2^)

**Table:**

	*U*^11^	*U*^22^	*U*^33^	*U*^12^	*U*^13^	*U*^23^
C1A	0.023 (5)	0.024 (3)	0.010 (3)	−0.008 (4)	0.004 (4)	−0.001 (2)
C2A	0.028 (3)	0.033 (3)	0.014 (2)	−0.007 (2)	0.002 (2)	0.0017 (19)
C3A	0.040 (4)	0.041 (4)	0.020 (3)	−0.004 (3)	0.006 (3)	−0.005 (3)
C4A	0.054 (4)	0.040 (3)	0.015 (2)	0.007 (3)	0.011 (3)	0.005 (2)
C5A	0.065 (7)	0.030 (4)	0.024 (3)	0.000 (4)	0.032 (5)	0.002 (2)
C6A	0.036 (4)	0.021 (3)	0.019 (4)	0.001 (3)	0.019 (3)	0.003 (2)
C1B	0.012 (8)	0.039 (6)	0.012 (5)	−0.002 (7)	0.000 (7)	0.003 (4)
C2B	0.031 (5)	0.035 (5)	0.014 (4)	−0.005 (4)	0.010 (4)	−0.003 (3)
C3B	0.027 (5)	0.044 (6)	0.008 (4)	0.002 (4)	0.005 (4)	−0.004 (4)
C4B	0.043 (6)	0.056 (6)	0.017 (4)	0.002 (5)	0.014 (4)	0.004 (4)
C5B	0.057 (11)	0.042 (7)	0.033 (6)	0.008 (7)	0.037 (8)	0.008 (5)
C6B	0.043 (6)	0.056 (6)	0.017 (4)	0.002 (5)	0.014 (4)	0.004 (4)
C11A	0.027 (2)	0.0199 (15)	0.019 (3)	−0.0022 (14)	0.0149 (19)	−0.0038 (15)
C16A	0.0252 (17)	0.0286 (17)	0.0296 (16)	−0.0017 (14)	0.0161 (15)	−0.0051 (14)
C15A	0.031 (2)	0.0288 (18)	0.030 (7)	0.0092 (19)	0.018 (3)	0.002 (4)
C14A	0.049 (4)	0.022 (3)	0.052 (6)	0.007 (3)	0.029 (5)	−0.001 (4)
C13A	0.034 (4)	0.025 (3)	0.070 (9)	−0.006 (3)	0.018 (4)	−0.012 (4)
C12A	0.025 (3)	0.025 (3)	0.046 (6)	0.000 (3)	0.011 (4)	−0.010 (4)
C11B	0.027 (2)	0.0199 (15)	0.019 (3)	−0.0022 (14)	0.0149 (19)	−0.0038 (15)
C12B	0.032 (5)	0.017 (4)	0.024 (6)	−0.001 (3)	0.019 (5)	−0.003 (4)
C13B	0.035 (5)	0.023 (4)	0.030 (7)	−0.006 (4)	0.017 (5)	−0.012 (4)
C14B	0.038 (6)	0.016 (4)	0.039 (8)	−0.001 (4)	0.027 (6)	−0.006 (5)
C15B	0.031 (2)	0.0288 (18)	0.030 (7)	0.0092 (19)	0.018 (3)	0.002 (4)
C16B	0.0252 (17)	0.0286 (17)	0.0296 (16)	−0.0017 (14)	0.0161 (15)	−0.0051 (14)
C21	0.0253 (15)	0.0255 (14)	0.0212 (14)	0.0019 (12)	0.0163 (13)	0.0016 (11)
C22	0.0345 (18)	0.0308 (16)	0.0248 (15)	−0.0004 (14)	0.0203 (14)	−0.0038 (12)
C23	0.039 (2)	0.0436 (18)	0.0295 (17)	0.0050 (16)	0.0266 (16)	0.0002 (14)
C24	0.0257 (17)	0.042 (2)	0.0318 (17)	0.0063 (14)	0.0198 (15)	0.0111 (14)
C25	0.0208 (15)	0.0318 (16)	0.0275 (15)	−0.0008 (13)	0.0116 (13)	0.0047 (13)
C26	0.0255 (16)	0.0252 (15)	0.0227 (14)	0.0006 (12)	0.0140 (13)	0.0008 (11)
C31	0.0173 (13)	0.0249 (14)	0.0163 (12)	−0.0023 (11)	0.0067 (11)	0.0010 (11)
C32	0.0370 (18)	0.0249 (15)	0.0320 (16)	−0.0060 (13)	0.0241 (15)	−0.0056 (13)
C33	0.044 (2)	0.0319 (17)	0.045 (2)	−0.0157 (16)	0.0294 (18)	−0.0077 (15)
C34	0.0286 (17)	0.0397 (19)	0.0355 (18)	−0.0063 (14)	0.0201 (15)	0.0035 (14)
C35	0.0228 (15)	0.0404 (19)	0.0280 (16)	0.0037 (14)	0.0147 (14)	0.0039 (13)
C36	0.0191 (14)	0.0239 (15)	0.0234 (14)	0.0027 (12)	0.0092 (12)	0.0015 (12)
C41	0.0284 (15)	0.0186 (13)	0.0186 (13)	0.0023 (12)	0.0137 (12)	0.0007 (10)
C42	0.0322 (17)	0.0258 (15)	0.0198 (14)	0.0026 (13)	0.0152 (13)	0.0017 (11)
C43	0.0397 (19)	0.0331 (17)	0.0240 (15)	0.0123 (15)	0.0186 (15)	0.0094 (13)
C44	0.055 (2)	0.0254 (17)	0.045 (2)	0.0091 (16)	0.0297 (19)	0.0127 (15)
C45	0.047 (2)	0.0271 (18)	0.056 (2)	−0.0065 (16)	0.024 (2)	0.0057 (16)
C46	0.0327 (18)	0.0223 (15)	0.0345 (17)	−0.0024 (13)	0.0142 (15)	0.0014 (13)
N1	0.0277 (13)	0.0195 (11)	0.0122 (11)	−0.0019 (10)	0.0094 (10)	−0.0025 (9)
F1	0.0658 (15)	0.0295 (10)	0.0423 (12)	0.0081 (10)	0.0237 (11)	0.0069 (9)
F2	0.0586 (15)	0.0359 (11)	0.0416 (12)	−0.0169 (10)	0.0104 (11)	−0.0100 (9)
F3	0.0660 (17)	0.098 (2)	0.0795 (18)	0.0130 (15)	0.0571 (16)	−0.0007 (16)
F4	0.0587 (15)	0.0308 (11)	0.0434 (12)	0.0029 (10)	0.0068 (11)	0.0053 (9)
F5	0.0654 (16)	0.0408 (13)	0.0424 (13)	−0.0176 (11)	0.0036 (12)	−0.0055 (10)
F6	0.0680 (17)	0.105 (2)	0.0836 (19)	0.0132 (16)	0.0631 (16)	0.0188 (16)
P1	0.0247 (4)	0.0178 (3)	0.0151 (3)	−0.0015 (3)	0.0131 (3)	−0.0026 (3)
P2	0.0191 (4)	0.0164 (3)	0.0139 (3)	−0.0011 (3)	0.0079 (3)	−0.0012 (3)
P3	0.0242 (4)	0.0221 (4)	0.0284 (4)	−0.0023 (3)	0.0136 (3)	−0.0016 (3)
Pt1	0.01666 (8)	0.01500 (7)	0.01119 (7)	−0.00172 (6)	0.00838 (6)	−0.00207 (5)
Cl1A	0.129 (3)	0.0727 (19)	0.105 (2)	−0.0276 (17)	0.087 (2)	−0.0308 (16)
Cl2A	0.087 (2)	0.0546 (13)	0.0543 (13)	0.0015 (13)	0.0400 (14)	0.0013 (10)
C01A	0.122 (10)	0.081 (7)	0.066 (6)	−0.027 (7)	0.055 (7)	−0.025 (5)
Cl2B	0.0664 (18)	0.0422 (12)	0.0650 (15)	−0.0022 (11)	0.0447 (14)	−0.0095 (10)
C01B	0.048 (5)	0.041 (5)	0.041 (5)	0.007 (4)	0.014 (4)	0.010 (4)

### Geometric parameters (Å, °)

**Table:**

C1A—N1	1.480 (12)	C15B—H15B	0.95
C1A—C6A	1.50 (2)	C16B—H16B	0.95
C1A—C2A	1.533 (10)	C21—C26	1.393 (4)
C1A—H1A	1.00	C21—C22	1.400 (4)
C2A—C3A	1.529 (7)	C21—P1	1.799 (3)
C2A—H2A1	0.99	C22—C23	1.387 (4)
C2A—H2A2	0.99	C22—H22	0.95
C3A—C4A	1.540 (9)	C23—C24	1.386 (5)
C3A—H3A1	0.99	C23—H23	0.95
C3A—H3A2	0.99	C24—C25	1.386 (4)
C4A—C5A	1.508 (12)	C24—H24	0.95
C4A—H4A1	0.99	C25—C26	1.384 (4)
C4A—H4A2	0.99	C25—H25	0.95
C5A—C6A	1.51 (2)	C26—H26	0.95
C5A—H5A1	0.99	C31—C32	1.386 (4)
C5A—H5A2	0.99	C31—C36	1.398 (4)
C6A—H6A1	0.99	C31—P2	1.804 (3)
C6A—H6A2	0.99	C32—C33	1.389 (4)
C1B—C2B	1.46 (2)	C32—H32	0.95
C1B—N1	1.56 (2)	C33—C34	1.381 (5)
C1B—C6B	1.60 (5)	C33—H33	0.95
C1B—H1B	1.00	C34—C35	1.382 (4)
C2B—C3B	1.528 (11)	C34—H34	0.95
C2B—H2B1	0.99	C35—C36	1.385 (4)
C2B—H2B2	0.99	C35—H35	0.95
C3B—C4B	1.514 (15)	C36—H36	0.95
C3B—H3B1	0.99	C41—C46	1.385 (4)
C3B—H3B2	0.99	C41—C42	1.396 (4)
C4B—C5B	1.52 (2)	C41—P2	1.794 (3)
C5B—C6B	1.57 (5)	C42—C43	1.379 (4)
C5B—H5B1	0.99	C42—H42	0.95
C5B—H5B2	0.99	C43—C44	1.370 (5)
C6B—H6B1	0.99	C43—H43	0.95
C6B—H6B2	0.99	C44—C45	1.378 (5)
C11A—C12A	1.38 (3)	C44—H44	0.95
C11A—C16A	1.40 (3)	C45—C46	1.382 (5)
C11A—P1	1.811 (16)	C45—H45	0.95
C16A—C15A	1.385 (15)	C46—H46	0.95
C16A—H16A	0.95	N1—P1	1.701 (3)
C15A—C14A	1.384 (15)	N1—P2	1.706 (2)
C15A—H15A	0.95	F1—P3	1.580 (2)
C14A—C13A	1.378 (12)	F2—P3	1.593 (2)
C14A—H14A	0.95	F3—P3	1.591 (2)
C13A—C12A	1.387 (12)	F4—P3	1.598 (2)
C13A—H13A	0.95	F5—P3	1.590 (2)
C12A—H12A	0.95	F6—P3	1.564 (2)
C11B—C16B	1.37 (4)	Pt1—P1	2.2918 (9)
C11B—C12B	1.40 (4)	Pt1—P2	2.2999 (9)
C11B—P1	1.79 (3)	Cl1A—C01A	1.747 (11)
C12B—C13B	1.383 (15)	Cl2A—C01A	1.765 (11)
C12B—H12B	0.95	C01A—H01A	0.99
C13B—C14B	1.381 (15)	C01A—H01B	0.99
C13B—H13B	0.95	Cl1B—C01B	1.760 (8)
C14B—C15B	1.38 (2)	Cl2B—C01B	1.757 (9)
C14B—H14B	0.95	C01B—H01C	0.99
C15B—C16B	1.39 (2)	C01B—H01D	0.99
			
N1—C1A—C6A	116.4 (11)	C22—C21—P1	120.9 (2)
N1—C1A—C2A	109.4 (7)	C23—C22—C21	119.7 (3)
C6A—C1A—C2A	109.6 (11)	C23—C22—H22	120.1
N1—C1A—H1A	107	C21—C22—H22	120.1
C6A—C1A—H1A	107	C24—C23—C22	120.2 (3)
C2A—C1A—H1A	107	C24—C23—H23	119.9
C3A—C2A—C1A	110.4 (6)	C22—C23—H23	119.9
C3A—C2A—H2A1	109.6	C23—C24—C25	120.4 (3)
C1A—C2A—H2A1	109.6	C23—C24—H24	119.8
C3A—C2A—H2A2	109.6	C25—C24—H24	119.8
C1A—C2A—H2A2	109.6	C26—C25—C24	119.8 (3)
H2A1—C2A—H2A2	108.1	C26—C25—H25	120.1
C2A—C3A—C4A	111.9 (5)	C24—C25—H25	120.1
C2A—C3A—H3A1	109.2	C25—C26—C21	120.5 (3)
C4A—C3A—H3A1	109.2	C25—C26—H26	119.8
C2A—C3A—H3A2	109.2	C21—C26—H26	119.8
C4A—C3A—H3A2	109.2	C32—C31—C36	119.4 (3)
H3A1—C3A—H3A2	107.9	C32—C31—P2	120.6 (2)
C5A—C4A—C3A	111.2 (6)	C36—C31—P2	119.4 (2)
C5A—C4A—H4A1	109.4	C31—C32—C33	120.3 (3)
C3A—C4A—H4A1	109.4	C31—C32—H32	119.8
C5A—C4A—H4A2	109.4	C33—C32—H32	119.8
C3A—C4A—H4A2	109.4	C34—C33—C32	119.9 (3)
H4A1—C4A—H4A2	108	C34—C33—H33	120
C4A—C5A—C6A	109.7 (9)	C32—C33—H33	120
C4A—C5A—H5A1	109.7	C33—C34—C35	120.2 (3)
C6A—C5A—H5A1	109.7	C33—C34—H34	119.9
C4A—C5A—H5A2	109.7	C35—C34—H34	119.9
C6A—C5A—H5A2	109.7	C34—C35—C36	120.2 (3)
H5A1—C5A—H5A2	108.2	C34—C35—H35	119.9
C1A—C6A—C5A	114.3 (14)	C36—C35—H35	119.9
C1A—C6A—H6A1	108.7	C35—C36—C31	119.9 (3)
C5A—C6A—H6A1	108.7	C35—C36—H36	120
C1A—C6A—H6A2	108.7	C31—C36—H36	120
C5A—C6A—H6A2	108.7	C46—C41—C42	119.4 (3)
H6A1—C6A—H6A2	107.6	C46—C41—P2	118.3 (2)
C2B—C1B—N1	113.5 (14)	C42—C41—P2	122.2 (2)
C2B—C1B—C6B	116 (2)	C43—C42—C41	120.1 (3)
N1—C1B—C6B	103 (2)	C43—C42—H42	119.9
C2B—C1B—H1B	107.9	C41—C42—H42	119.9
N1—C1B—H1B	107.9	C44—C43—C42	120.0 (3)
C6B—C1B—H1B	107.9	C44—C43—H43	120
C1B—C2B—C3B	109.1 (11)	C42—C43—H43	120
C1B—C2B—H2B1	109.9	C43—C44—C45	120.3 (3)
C3B—C2B—H2B1	109.9	C43—C44—H44	119.8
C1B—C2B—H2B2	109.9	C45—C44—H44	119.8
C3B—C2B—H2B2	109.9	C44—C45—C46	120.3 (3)
H2B1—C2B—H2B2	108.3	C44—C45—H45	119.8
C4B—C3B—C2B	113.4 (8)	C46—C45—H45	119.8
C4B—C3B—H3B1	108.9	C45—C46—C41	119.8 (3)
C2B—C3B—H3B1	108.9	C45—C46—H46	120.1
C4B—C3B—H3B2	108.9	C41—C46—H46	120.1
C2B—C3B—H3B2	108.9	C1A—N1—P1	120.1 (4)
H3B1—C3B—H3B2	107.7	C1B—N1—P1	137.3 (6)
C3B—C4B—C5B	110.2 (9)	C1A—N1—P2	134.0 (4)
C4B—C5B—C6B	115.4 (19)	C1B—N1—P2	119.3 (7)
C4B—C5B—H5B1	108.4	P1—N1—P2	102.58 (12)
C6B—C5B—H5B1	108.4	N1—P1—C11B	110.8 (17)
C4B—C5B—H5B2	108.4	N1—P1—C21	111.32 (13)
C6B—C5B—H5B2	108.4	C11B—P1—C21	102.4 (13)
H5B1—C5B—H5B2	107.5	N1—P1—C11A	110.5 (11)
C5B—C6B—C1B	103 (3)	C21—P1—C11A	106.0 (9)
C5B—C6B—H6B1	111.1	N1—P1—Pt1	93.47 (8)
C1B—C6B—H6B1	111.1	C11B—P1—Pt1	121.4 (10)
C5B—C6B—H6B2	111.1	C21—P1—Pt1	117.31 (10)
C1B—C6B—H6B2	111.1	C11A—P1—Pt1	117.7 (6)
H6B1—C6B—H6B2	109	N1—P2—C41	110.98 (12)
C12A—C11A—C16A	119.5 (11)	N1—P2—C31	110.86 (13)
C12A—C11A—P1	123 (2)	C41—P2—C31	107.74 (14)
C16A—C11A—P1	116.8 (19)	N1—P2—Pt1	93.08 (9)
C15A—C16A—C11A	120.6 (15)	C41—P2—Pt1	117.31 (10)
C15A—C16A—H16A	119.7	C31—P2—Pt1	116.09 (10)
C11A—C16A—H16A	119.7	F6—P3—F1	91.14 (14)
C14A—C15A—C16A	119.1 (13)	F6—P3—F5	90.24 (16)
C14A—C15A—H15A	120.4	F1—P3—F5	90.30 (12)
C16A—C15A—H15A	120.4	F6—P3—F3	179.34 (18)
C13A—C14A—C15A	120.3 (9)	F1—P3—F3	89.15 (14)
C13A—C14A—H14A	119.9	F5—P3—F3	89.16 (16)
C15A—C14A—H14A	119.9	F6—P3—F2	91.09 (16)
C14A—C13A—C12A	120.7 (8)	F1—P3—F2	90.39 (12)
C14A—C13A—H13A	119.7	F5—P3—F2	178.49 (15)
C12A—C13A—H13A	119.7	F3—P3—F2	89.51 (15)
C11A—C12A—C13A	119.7 (12)	F6—P3—F4	90.00 (15)
C11A—C12A—H12A	120.2	F1—P3—F4	178.76 (14)
C13A—C12A—H12A	120.2	F5—P3—F4	90.20 (12)
C16B—C11B—C12B	119.3 (16)	F3—P3—F4	89.72 (15)
C16B—C11B—P1	120 (3)	F2—P3—F4	89.08 (12)
C12B—C11B—P1	121 (3)	P1^i^—Pt1—P1	180.0000 (10)
C13B—C12B—C11B	120.5 (18)	P1^i^—Pt1—P2^i^	70.76 (3)
C13B—C12B—H12B	119.7	P1—Pt1—P2^i^	109.24 (3)
C11B—C12B—H12B	119.7	P1^i^—Pt1—P2	109.24 (3)
C14B—C13B—C12B	119.0 (10)	P1—Pt1—P2	70.76 (3)
C14B—C13B—H13B	120.5	P2^i^—Pt1—P2	180
C12B—C13B—H13B	120.5	Cl1A—C01A—Cl2A	111.5 (5)
C15B—C14B—C13B	120.9 (14)	Cl1A—C01A—H01A	109.3
C15B—C14B—H14B	119.5	Cl2A—C01A—H01A	109.3
C13B—C14B—H14B	119.5	Cl1A—C01A—H01B	109.3
C14B—C15B—C16B	120 (2)	Cl2A—C01A—H01B	109.3
C14B—C15B—H15B	120.1	H01A—C01A—H01B	108
C16B—C15B—H15B	120.1	Cl2B—C01B—Cl1B	110.9 (5)
C11B—C16B—C15B	120 (2)	Cl2B—C01B—H01C	109.5
C11B—C16B—H16B	119.8	Cl1B—C01B—H01C	109.5
C15B—C16B—H16B	119.8	Cl2B—C01B—H01D	109.5
C26—C21—C22	119.5 (3)	Cl1B—C01B—H01D	109.5
C26—C21—P1	119.6 (2)	H01C—C01B—H01D	108.1
			
N1—C1A—C2A—C3A	176.4 (6)	C1A—N1—P1—C11B	69.5 (12)
C6A—C1A—C2A—C3A	−54.8 (12)	C1B—N1—P1—C11B	62.4 (16)
C1A—C2A—C3A—C4A	55.2 (8)	P2—N1—P1—C11B	−128.4 (11)
C2A—C3A—C4A—C5A	−55.4 (8)	C1A—N1—P1—C21	−43.8 (5)
C3A—C4A—C5A—C6A	54.1 (10)	C1B—N1—P1—C21	−50.9 (12)
N1—C1A—C6A—C5A	−178.1 (9)	P2—N1—P1—C21	118.30 (14)
C2A—C1A—C6A—C5A	57.1 (15)	C1A—N1—P1—C11A	73.7 (9)
C4A—C5A—C6A—C1A	−56.8 (15)	C1B—N1—P1—C11A	66.6 (14)
N1—C1B—C2B—C3B	177.7 (9)	P2—N1—P1—C11A	−124.2 (7)
C6B—C1B—C2B—C3B	59 (2)	C1A—N1—P1—Pt1	−165.0 (5)
C1B—C2B—C3B—C4B	−55.3 (12)	C1B—N1—P1—Pt1	−172.1 (12)
C2B—C3B—C4B—C5B	53.7 (13)	P2—N1—P1—Pt1	−2.84 (11)
C3B—C4B—C5B—C6B	−55 (2)	C16B—C11B—P1—N1	−179.4 (19)
C4B—C5B—C6B—C1B	53 (3)	C12B—C11B—P1—N1	−1(2)
C2B—C1B—C6B—C5B	−56 (3)	C16B—C11B—P1—C21	−61 (2)
N1—C1B—C6B—C5B	179.2 (18)	C12B—C11B—P1—C21	118 (2)
C12A—C11A—C16A—C15A	0(2)	C16B—C11B—P1—Pt1	73 (2)
P1—C11A—C16A—C15A	−172.2 (17)	C12B—C11B—P1—Pt1	−108.6 (18)
C11A—C16A—C15A—C14A	−2(2)	C26—C21—P1—N1	−96.5 (3)
C16A—C15A—C14A—C13A	2.3 (18)	C22—C21—P1—N1	81.8 (3)
C15A—C14A—C13A—C12A	0.1 (16)	C26—C21—P1—C11B	145.0 (15)
C16A—C11A—C12A—C13A	3(2)	C22—C21—P1—C11B	−36.7 (15)
P1—C11A—C12A—C13A	174.0 (9)	C26—C21—P1—C11A	143.3 (10)
C14A—C13A—C12A—C11A	−2.6 (18)	C22—C21—P1—C11A	−38.4 (10)
C16B—C11B—C12B—C13B	0(2)	C26—C21—P1—Pt1	9.5 (3)
P1—C11B—C12B—C13B	−178.7 (16)	C22—C21—P1—Pt1	−172.2 (2)
C11B—C12B—C13B—C14B	−1.0 (14)	C12A—C11A—P1—N1	17.2 (18)
C12B—C13B—C14B—C15B	3(2)	C16A—C11A—P1—N1	−171.4 (12)
C13B—C14B—C15B—C16B	−4(3)	C12A—C11A—P1—C21	138.0 (15)
C12B—C11B—C16B—C15B	−1(3)	C16A—C11A—P1—C21	−50.7 (13)
P1—C11B—C16B—C15B	178 (3)	C12A—C11A—P1—Pt1	−88.4 (18)
C14B—C15B—C16B—C11B	3(4)	C16A—C11A—P1—Pt1	83.0 (13)
C26—C21—C22—C23	0.8 (5)	C1A—N1—P2—C41	40.3 (6)
P1—C21—C22—C23	−177.4 (3)	C1B—N1—P2—C41	53.6 (9)
C21—C22—C23—C24	−0.9 (5)	P1—N1—P2—C41	−118.02 (14)
C22—C23—C24—C25	−0.1 (5)	C1A—N1—P2—C31	−79.4 (6)
C23—C24—C25—C26	1.3 (5)	C1B—N1—P2—C31	−66.1 (9)
C24—C25—C26—C21	−1.4 (5)	P1—N1—P2—C31	122.29 (14)
C22—C21—C26—C25	0.3 (5)	C1A—N1—P2—Pt1	161.2 (6)
P1—C21—C26—C25	178.6 (2)	C1B—N1—P2—Pt1	174.5 (9)
C36—C31—C32—C33	−0.4 (5)	P1—N1—P2—Pt1	2.83 (11)
P2—C31—C32—C33	−171.3 (3)	C46—C41—P2—N1	86.4 (3)
C31—C32—C33—C34	1.3 (5)	C42—C41—P2—N1	−89.9 (3)
C32—C33—C34—C35	−0.9 (5)	C46—C41—P2—C31	−152.0 (2)
C33—C34—C35—C36	−0.4 (5)	C42—C41—P2—C31	31.6 (3)
C34—C35—C36—C31	1.3 (5)	C46—C41—P2—Pt1	−18.8 (3)
C32—C31—C36—C35	−0.9 (4)	C42—C41—P2—Pt1	164.8 (2)
P2—C31—C36—C35	170.1 (2)	C32—C31—P2—N1	−26.6 (3)
C46—C41—C42—C43	−1.2 (4)	C36—C31—P2—N1	162.4 (2)
P2—C41—C42—C43	175.1 (2)	C32—C31—P2—C41	−148.3 (2)
C41—C42—C43—C44	0.2 (5)	C36—C31—P2—C41	40.8 (3)
C42—C43—C44—C45	0.6 (5)	C32—C31—P2—Pt1	77.9 (3)
C43—C44—C45—C46	−0.5 (6)	C36—C31—P2—Pt1	−93.1 (2)
C44—C45—C46—C41	−0.4 (6)	N1—P1—Pt1—P2^i^	−177.82 (8)
C42—C41—C46—C45	1.3 (5)	C11B—P1—Pt1—P2^i^	−60.8 (19)
P2—C41—C46—C45	−175.2 (3)	C21—P1—Pt1—P2^i^	65.99 (12)
C6A—C1A—N1—C1B	−101 (4)	C11A—P1—Pt1—P2^i^	−62.5 (12)
C2A—C1A—N1—C1B	23 (3)	N1—P1—Pt1—P2	2.18 (8)
C6A—C1A—N1—P1	94.2 (10)	C11B—P1—Pt1—P2	119.2 (19)
C2A—C1A—N1—P1	−140.9 (5)	C21—P1—Pt1—P2	−114.01 (12)
C6A—C1A—N1—P2	−61.2 (11)	C11A—P1—Pt1—P2	117.5 (12)
C2A—C1A—N1—P2	63.7 (10)	N1—P2—Pt1—P1^i^	177.83 (8)
C2B—C1B—N1—C1A	−70 (4)	C41—P2—Pt1—P1^i^	−66.62 (11)
C6B—C1B—N1—C1A	57 (4)	C31—P2—Pt1—P1^i^	62.78 (11)
C2B—C1B—N1—P1	−49.5 (17)	N1—P2—Pt1—P1	−2.17 (8)
C6B—C1B—N1—P1	77 (2)	C41—P2—Pt1—P1	113.38 (11)
C2B—C1B—N1—P2	142.5 (8)	C31—P2—Pt1—P1	−117.22 (11)
C6B—C1B—N1—P2	−90.8 (17)		

Symmetry codes: (i) −*x*+1, −*y*+1, −*z*+1.

### Hydrogen-bond geometry (Å, °)

**Table:**

*D*—H···*A*	*D*—H	H···*A*	*D*···*A*	*D*—H···*A*
C12A—H12A···F2	0.95	2.45	3.186 (11)	134
C32—H32···F2	0.95	2.47	3.138 (4)	127
